# Skeletal and dental changes induced by the Flip-Lock Herbst appliance in the treatment of Angle’s class II division 1 malocclusion during active growth period: A preliminary study

**DOI:** 10.34172/joddd.2021.011

**Published:** 2021-02-13

**Authors:** Sushmitha R Iyer, Sridhar Premkumar, Mangaleswari Muruganandam

**Affiliations:** ^1^Department of Orthodontics & Dentofacial Orthopaedics, Chettinad Dental College and Research Institute, Kancheepuram, Tamil Nadu, India; ^2^Department of Orthodontics & Dentofacial Orthopaedics, Tamil Nadu Government Dental College and Hospital, Chennai, Tamil Nadu, India; ^3^Directorate of Medical and Rural Health Services, Madhurandagam Government Hospital, Chengalpattu, Tamil Nadu, India

**Keywords:** Class II, Functional treatment, Herbst appliance

## Abstract

**Background.** The Flip-Lock Herbst (TP Orthodontics Inc.) is a fixed functional appliance, a variant of the Herbst appliance, introduced by Miller. It is claimed to have better patient tolerance due to its increased freedom for the mandible’s lateral movements. There have been no studies on the flip lock Herbst till date. This study was undertaken to assess the efficiency of the Flip-Lock Herbst appliance in correcting Angle’s class II division 1 malocclusion.

**Methods.** Eight subjects in their active growth period with class II division 1 malocclusion due to a retrognathic mandible were included in the study. Standardized lateral cephalometric radiographs were used to evaluate skeletal and dental changes with the SO analysis. Paired samples t-test was used to assess statistical significance.

**Results.** Statistically significant increases in mandibular length (pg/OLp) and effective mandibular length (ar/OLp + pg/OLp) were observed. There was a significant maxillary restraining effect. Dental effects were significant and exhibited class II correction features except for the position of lower incisors within the mandible (ii/OLp - pg/OLp). Skeletal changes accounted for 61% and dental changes for 39% of the total treatment for molar correction. For overjet correction, skeletal changes contributed to 63% and dental changes to 37% of the total treatment.

**Conclusion.** The Flip-Lock Herbst appliance was efficient in correcting Angle’s class II division 1 malocclusion due to a retrognathic mandible. Both skeletal and dental changes were evident, with the former predominating (60:40).

## Introduction

Growth modification is typically carried out during the adolescent period, which is already rife with many social and developmental changes. The success of any treatment depends on patient compliance, which is difficult to predict and, to some extent, depends on the degree of discomfort and treatment duration.^1^ Fixed functional appliances (FFAs) place the onus of treatment on the orthodontist and have the advantage of being compliance-free. Furthermore, since they jump the bite continuously, they act full time and shorten treatment duration.^2-4^ Patient perception of treatment is an important factor, and this varies among the three types of FFAs: rigid, semi-rigid, and flexible.^5,6^ The Herbst appliance, introduced by Dr. Emil Herbst in 1909 and later reintroduced by Pancherz^7^ in 1979, is a type of rigid fixed functional appliance. It has shown consistent results in the correction of class II malocclusion. The disadvantages of the Herbst appliance include masticatory problems, soft tissue impingement, breakage or distortion of the appliance, bent rods, and loose or broken bands and screws.^7,8^

The Flip-Lock Herbst (TP Orthodontics Inc.) is a variant of the Herbst appliance, introduced by Miller,^9^ which uses ball joints as a locking mechanism. It is claimed to have better patient comfort and acceptance due to its increased freedom for lateral movements in the mandible, a lower breakage rate, and reduced chairside time.^9^

Although several studies on the Herbst appliance have shown its effectiveness in correcting class II malocclusion, there are no studies to date on the Flip-Lock Herbst appliance. Therefore, this preliminary study was undertaken to assess the efficiency of the Flip-Lock Herbst appliance in patients with Angle’s class II division 1 malocclusion due to a retrognathic mandible during the active growth period. The objective was to analyze the skeletal and dental changes in patients treated with the Flip-Lock Herbst appliance cumulatively and separately.

## Methods

A preliminary study was planned, and eight patients with class II division 1 malocclusion, who reported to the Department of Orthodontics and Dentofacial Orthopedics in one governmental dental college and hospital, were treated with the Flip-Lock Herbst appliance after obtaining ethical clearance from the Institutional Ethics Committee. The age of the subjects ranged from 12 to 15.8 years, with a mean of 13 years. The treatment duration lasted for 7.9 months on average, ranging from 6.1 to 10.3 months. The details of the patients are summarized in Table 1.

**Table 1:** Summary of details of patients treated for the study

	**1**	**2**	**3**	**4**	**5**	**6**	**7**	**8**
**Duration of treatment**	8 months	8 months	9 months	7 months	9 months	7 months	7 months	8 months
**Age**	12 years 4 months	12 years 3 months	15 years 1 month	13 years 5 months	13 years 9 months	13 years 2 months	12 years	12 years 10 months
**Sex**	Female	Female	Male	Female	Male	Male	Female	Male
**Hand wrist stage**	Stage 4	Stage 5	Stage 5	Stage 5	Stage 4	Stage 4	Stage 4	Stage 4

### Inclusion criteria

1. Patients willing to participate.
2. Permanent dentition with class II division 1 malocclusion.
3. Bilateral full cusp class II molar relationship.
4. Positive VTO (visual treatment objective) with mandibular advancement.
5. Overjet of 7‒9 mm.
6. Patients in active growth period (stage: fourth or fifth according to Björk,^10^ Grave and Brown method).^11^
7. Retrognathic mandible (SNB: 74‒77°; Nasion perpendicular to Pogonion; Co-Gn).
8. Orthognathic maxilla (SNA: 82±2°; Point A to Nasion perpendicular; Co-A).
9. Average growth pattern.

### Exclusion criteria

1. Patients with proclined lower incisors (IMPA: >110°)
2. Patients with prognathic maxilla
3. Patients with upper and lower incisor crowding
4. Presence with midline deviation
5. Previous history of orthodontic treatment
6. Previous history of trauma
7. Systemic diseases
8. Periodontal disorders

### Records

**Figure 1:**
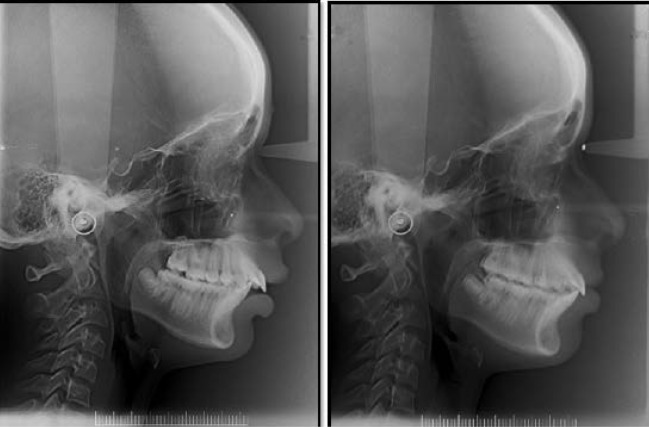
Standardized lateral cephalometric radiographs at T1 and T2.

**Figure 2:**
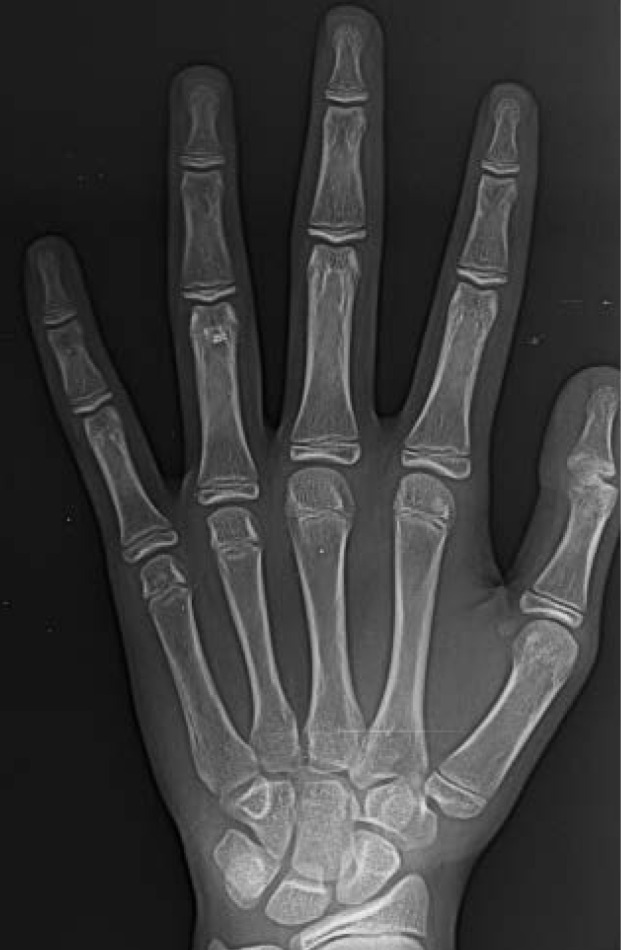
Hand wrist radiograph to determine growth status.

**Figure 3:**
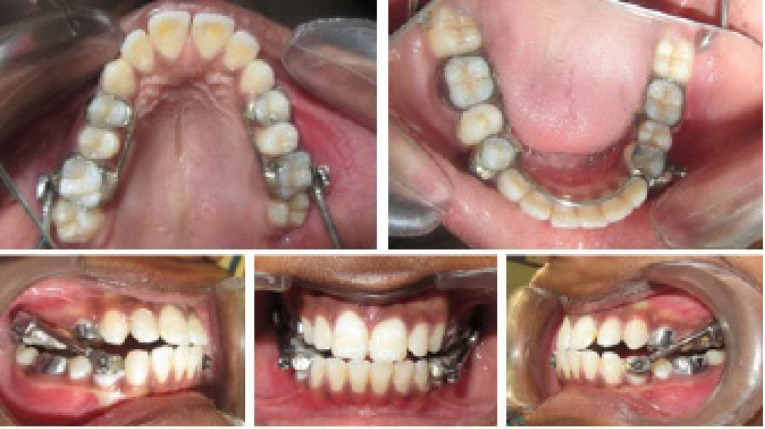
Intraoral photographs at T1, depicting partial anchorage in the upper arch and total anchorage in the lower arch.

**Figure 4:**
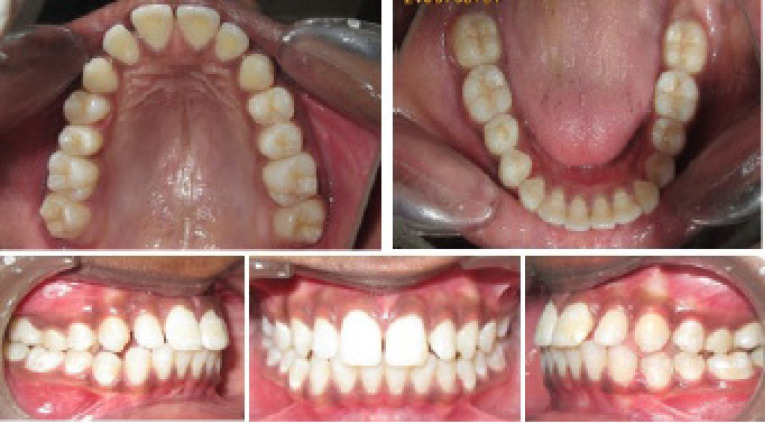
Intraoral photographs at T2, depicting the end of functional phase.

The following sets of records were taken at T1 (before the start of treatment) and T2 (after completion of functional therapy):

1. Standardized lateral cephalometric radiographs (Figure 1).
2. Hand wrist radiographs to assess skeletal maturity at T1 (Figure 2).
3. Intraoral photographs (Figures 3 and 4).

### Appliance design and bite jumping

**Figure 5:**
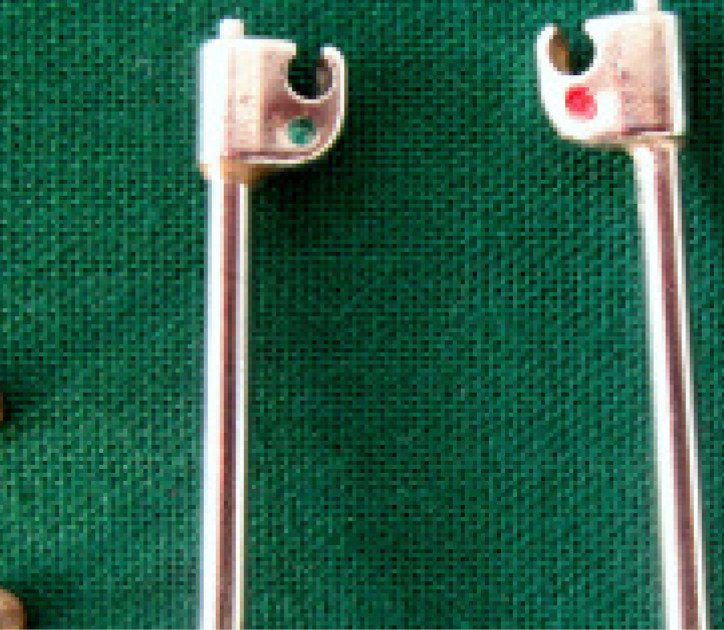
Components of the Flip-Lock Herbst appliance with the ball connectors, plungers, and scribe to distinguish red and left.

The Flip-Lock Herbst appliance (TP Orthodontics Inc.) consists of two ball connectors, a tube, and a plunger on each side^9^ (Figure 5). Upper first molars and first premolars were banded, and the anchorage was reinforced with a 0.032” stainless steel lingual wire soldered to the first molar and first premolar on each side (Figure 4).^7^ In the lower arch, first molars and first premolars were banded and stabilized with a 0.032” stainless steel lingual wire soldered to the first molar and first premolar on both sides. The ball joint connectors for the appliance were soldered on to the buccal surfaces of the bands on the upper first molars and lower first premolars. The framework was cemented to the upper and lower arches. The tube was connected to the upper ball joint member. Right and left sides were distinguished by red and green dots scribed on the upper head of the tube (Figure 5). The plunger length was measured and cut in accordance with the advancement needed to achieve class I molar relation. The plunger was inserted into the tube, and the patient was asked to advance the mandible so that the plunger end could be fitted onto the ball joint connector in the lower first premolar. The tubes and plungers were fitted onto their respective ball joint connectors, and the snap fit was established. For the first month, patients were reviewed once a week. From the next month onwards, they were reviewed once a month. Changes in molar relationships were monitored during the monthly reviews by removing the plunger and tube. When a class I molar relationship was achieved, the appliance was removed, and records for T2 were taken.

### Cephalometric analysis

**Figure 6:**
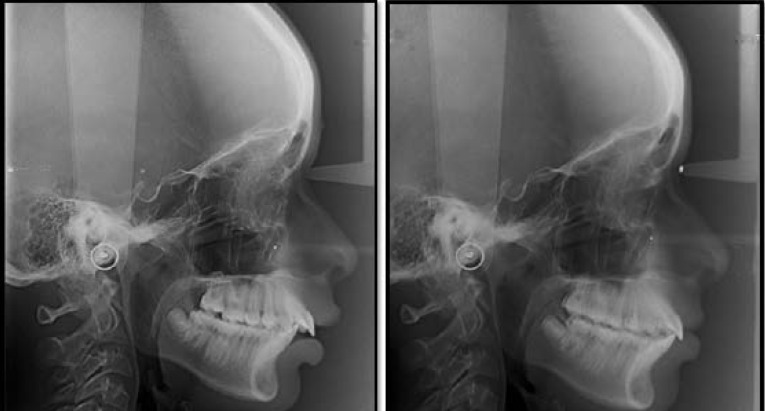
SO analysis^12^ to assess post-functional treatment changes with T1 tracing in black and T2 in red.

Skeletal and dental effects were assessed through Pancherz’s SO analysis (Figure 6).^12^

The pre-functional treatment changes (T1) were traced in black and the post-functional changes (T2) in red.

Reference planes for the analysis were: NSL (anterior cranial base); OL (occlusal line); MP (mandibular plane), and OLp (occlusal line perpendicular).

The OL and the OLp from T1 lateral cephalogram were used as a reference plane and transferred to T2 by superimposing the tracings on the NSL with S as a registration point.

The following landmarks were identified, and the parameters were measured.

- ii and is: The incisal tips of the lower and upper central incisor, respectively.
- mi and ms: The contact point of the mesial surface of the lower permanent first molar and upper permanent first molar, respectively.
- Ss: The deepest point in the concavity of the upper alveolar process.
- Pg: The most anterior point on the chin.
- Ar: The intersection of the posterior ramal border with the inferior border of the posterior cranial base.
- ss/OLp and pg/OLp: The positions of the maxilla and mandible in the sagittal plane, respectively.
- ar/OLp: The position of the condyle.
- pg/OLp+ar/OLp: Effective mandibular length.
- NSL/MP: Growth pattern of the lower jaw.
- ii/OLp and is/OLp: The position of the lower and upper central incisors, respectively.
- is/OLp-ii/OLp: Overjet.
- mi/OLp and ms/OLp: The positions of the lower and upper first molars.
- ms/OLp-mi/OLp: Molar relationship.
- is/OLp-ss/OLp: The position of the upper central incisor within the maxilla.
- ii/OLp-pg/OLp: The position of the lower central incisor within the mandible.
- ms/OLp-ss/OLp: The position of the upper molar within the maxilla.
- mi/OLp-pg/OLp: The position of the lower molar within the mandible.

Pre-treatment (T1) and post-treatment (T2) values were calculated for skeletal and dental cephalometric variables and tabulated (Table 2).

**Table 2:** Pre- and post-functional treatment values of skeletal and dental parameters

**Variables/** **Subjects**	**1**		**2**		**3**		**4**		**5**		**6**		**7**		**8**	
	**T1**	**T2**	**T1**	**T2**	**T1**	**T2**	**T1**	**T2**	**T1**	**T2**	**T1**	**T2**	**T1**	**T2**	**T1**	**T2**
**Skeletal**																
SNA	82	80	83	82	82	81	80	80	82	82	81.5	81	83	81	81	81
SNB	74	78	76	78	76	77.5	75	78	75	78.5	76.5	78	75	79	76	78
ANB	8	2	7	4	6	3.5	5	2	7	3.5	5	3	8	2	5	3
ss/OLp	72	69	72	70	73	71	70	70	72.5	69.5	73	72	73	70	73.5	71
pg/OLp	72	73	71	71.5	70	71.5	69	71	72	73.5	73	73.5	73	74	70	72.5
ar/OLp	10	9	9	9	10	10	10	10	9	9.5	9	8.5	10	9.5	9	9
pg/OLp+ar/OLp	82	82	80	80.5	80	81.5	79	81	81	83	82	82	83	83.5	79	81.5
NSL/MP	32	31	29	29	26	24	32	32	29	27	30	29.5	30	30	32	30.5
**Dental**																
is/OLp	84	79	85	79	84	80	81.5	79.5	83.5	79	82	80	85	80	83	81
ii/OLp	76	76	77	77	77	77	74	76	76	77	73	78	77	77	75.5	78
is/OLp-ii/OLp	8	3	8	2	7	3	7.5	3	7.5	2	5.5	2	8	3	7.5	3
ms/OLp	52	49	52.5	48	53	51.5	53	51.5	53	49	49	44.5	53	50	51	48
mi/OLp	50	52	50	52.5	51	53	50	54	50	52.5	45	48	50	53	48	51
ms/OLp-mi/OLp	2	-3	2.5	-4.5	2	-1.5	3	-2.5	3	-3.5	4	-3.5	3	-3	3	-3
is/OLp-ss/OLp	12	10	13	9	11	9	11.5	9.5	11	9.5	9	8	12	10	9.5	10
ii/OLp-pg/OLp	4	3	6	5.5	7	6.5	5	5	4	3.5	3.5	4.5	4	3	5.5	5.5
ms/OLp-ss/OLp	20	20	19.5	22	20	19.5	17	18.5	19.5	24.5	24	27.5	20	20	22.5	23
mi/OLp-pg/OLp	22	21	21	19.5	19	18.5	19	17	22	21	28	25	23	21	22	21.5

SO Analysis: Refer to Figure 6 for an explanation about the variables.

### Statistical analysis

The results of normality tests Kolmogorov-Smirnov and Shapiro-Wilks revealed that the variables followed a normal distribution. Therefore, to analyze the data, parametric methods were applied. To compare the mean values between pre-treatment and post-treatment periods, a paired-samples t-test was applied. To analyze the data, SPSS (IBM SPSS Statistics for Windows, version 22.0, Armonk, NY: IBM Corp. Released 2013) was used. The significance level was fixed at 5% (α = 0.05).

## Results

### Skeletal effects

There was a statistically significant increase in mandibular length measurement pg/OLp by 1.3 mm (*P*= 0.001) and effective mandibular length measurement pg/OLp+ar/OLp by 1.1 mm (*P*= 0.015). SNB showed a highly significant increase of 2.7° (*P*< 0.001). Maxillary position (SNA and ss/OLp) and ANB showed a statistically significant decrease. The mandibular plane underwent small but significant counterclockwise rotation by 0.9° (Table 3).

**Table 3:** Paired-samples t-test to compare mean values of skeletal parameters between pre- and post-treatment periods

**Variables**		**N**	**Mean**	**SD**	***t*** **-value**	***P *** **value**
SNA	Pre	8	81.813	0.9978	2.728	0.029
	Post	8	81.000	0.7559		
SNB	Pre	8	75.438	0.8210	7.124	<0.001
	Post	8	78.125	0.4432		
ANB	Pre	8	6.375	1.3025	6.089	<0.001
	Post	8	2.875	0.7906		
ss/Olp	Pre	8	72.375	1.0938	5.384	0.001
	Post	8	70.313	0.9613		
pg/OLp	Pre	8	71.250	1.4880	5.274	0.001
	Post	8	72.563	1.1160		
ar/OLp	Pre	8	9.500	0.5345	1.158	0.285
	Post	8	9.313	0.5303		
pg/OLp+ar/OLp	Pre	8	80.750	1.4880	3.211	0.015
	Post	8	81.875	0.9910		

Statistically significant increase in mandibular length (pg/OLp) and effective mandibular length (ar/OLp + pg/OLp).

### Dental effects

There was a highly significant reduction in overjet (*P*< 0.001) by 5 mm, with molar relationship correction (ms/OLp-mi/OLp). The upper molar (ms/OLp-ss/OLp) moved distally by 1.5 mm within the upper jaw (P=0.058), and the lower molar moved mesially within the lower jaw by 1.5 mm (*P*= 0.002). The maxillary incisor moved palatally (*P*< 0.001) by 1.8 mm. The position of the lower incisor was unchanged (Table 4).

**Table 4:** Paired-samples *t* test to compare mean values of dental parameters between pre- and post-treatment periods

**Variables**		**N**	**Mean**	**SD**	**t-value**	***P*** ** value**
is/OLp	Pre	8	83.500	1.2817	6.730	<0.001
	Post	8	79.688	0.7039		
ii/OLp	Pre	8	75.688	1.4865	2.072	0.077
	Post	8	77.000	0.7559		
is/OLp-ii/OLp	Pre	8	7.375	0.8345	16.756	<0.001
	Post	8	2.625	0.5175		
ms/OLp	Pre	8	52.063	1.4252	7.442	<0.001
	Post	8	48.938	2.2589		
mi/OLp	Pre	8	49.250	1.9086	11.881	<0.001
	Post	8	52.000	1.8323		
ms/OLp-mi/OLp	Pre	8	2.813	0.6512	13.332	<0.001
	Post	8	-3.063	0.8634		
is/OLp-ss/OLp	Pre	8	11.125	1.3296	3.949	0.006
	Post	8	9.375	0.6944		
ii/OLp-pg/OLp	Pre	8	4.875	1.2174	1.357	0.217
	Post	8	4.563	1.2939		
ms/OLp-ss/OLp	Pre	8	20.313	2.1034	2.262	0.058
	Post	8	21.875	3.0208		
mi/OLp-pg/OLp	Pre	8	22.000	2.8284	4.709	0.002
	Post	8	20.563	2.3670		

Overall, the dental effects were significant and favorable towards class II correction. The position of lower incisor within mandible (ii/OLp-pg/OLp) showed no significant changes.

## Discussion

The Flip-Lock Herbst appliance has a ball joint instead of screws, which connects the upper and lower molars. The proposed advantages of Flip-Lock, as quoted by the company, include an increased range of lateral movements, less bulk, and increased comfort for the patient.^9^ Patient perception of treatment, though overlooked, is an important factor in treatment success.^6^ In this study, only patients who volunteered for functional therapy, were selected.

Eight patients were selected consecutively, who fulfilled the inclusion and exclusion criteria. Consecutive selection of samples is a better alternative than other non-randomized trial designs.^13^ Skeletal criteria for selection was an orthognathic maxilla (as assessed by cephalometric variables SNA, Point A to Nasion perpendicular, and Co-A) and a retrognathic mandible (assessed by SNB, Nasion perpendicular to Pogonion, Co-Gn). Patients with orthognathic maxilla were included so that the effect of the appliance could be primarily assessed on the retrognathic mandible. Cases with mild to moderate mandibular retrognathism (SNB value of 74‒77°) were selected. The skeletal criteria reflect the regard for the phenotype of class II malocclusion,^14^ herein mandibular retrognathism. The dental criteria for selection (permanent dentition with no crowding in the upper and lower arches) allowed to directly start the functional phase without pre-functional fixed appliance treatment, which is the accepted norm when using an FFA. Subjects with overjet within the range of 7‒9 mm were included to have a uniform protocol of single-step advancement.

The anchorage design for the appliance consisted of total anchorage in the lower arch by the inclusion of teeth from the first molar on one side to the contralateral side in the lower arch, with partial anchorage in the upper arch by the inclusion of the first premolar to the first molar on each side (Figure 3). Achievement of class I molar relationship marked the end of the functional phase, and a change in the molar relationship was assessed easily by removing the tubes and plunger. Since it works by snap fit over the ball joints, removal and insertion are quite easy.

FJO literature is laden with controversies, with some studies showing promising results,^15-17^ inadequate effects,^18,19^ or partial effects.^20^ These differences can be partly attributed to the skeletal maturity at the time the treatment was instituted.^21^ Hence, this study was performed at or slightly before the pubertal growth spurt. The use of a reliable skeletal maturity indicator is essential. Here, skeletal maturity was assessed with HWR (hand wrist radiograph) by Björk and Grave and Brown technique.^10,11^ Accordingly, patients in stage 4 and 5 were selected (Figure 2).

Skeletal and dental changes were appraised through the SO analysis (sagittal occlusal analysis) developed by Pancherz (Figure 6).^12^ This analysis was carried out in addition to traditional jaw base parameters like SNA, SNB, and ANB. The SO analysis also facilitates a comparison between the present study on the Flip-Lock Herbst appliance and previous studies on the Herbst appliance.

The effects of Herbst appliance on the maxillary jaw has been documented as a headgear effect with the tipping of the palatal plane and intrusion and distal movement of molars, with no change in the sagittal maxillary position.^12,23^ In the present study, only class II malocclusion due to the orthognathic maxilla and retrognathic mandible was included. Overall, a maxillary restraining effect was appreciable. This effect was more pronounced than a previous study, which demonstrated a maxillary restraint with an SNA reduction of 0.5 degrees.^12^ The difference might be attributed to the difference in phenotype of the skeletal malocclusion. This study was carried out with strict inclusion criteria of the orthognathic maxilla. The effect of FFA on patients with prognathic maxilla versus orthognathic maxilla should be distinguished.

The changes in the position of the mandible were assessed by SNB and pg/OLp values, both of which showed significant increases (*P*< 0.001 and *P*= 0.001, respectively), consistent with previous studies.^12,22,24,25^ A small but significant counterclockwise (ccw) rotation from 30° to 29.1° in the mandibular plane was observed comparable to previous studies.^12,26,27^ Changes in the mandible position with functional therapy can be due to the sum of all changes, such as a positional change from correction of functional retrusion, anterior relocation of the fossa, and accompanying condylar growth in the sagittal direction, dual bite, or an actual increase in mandibular length. The effective mandibular length as a sum of positional changes and length changes is a better alternative than other linear measurements. In this study, the effective mandibular length (pg/OLp+ar/OLp) increased by 1.1 mm. This is a mean value and inter-individual variation existed in the changes ranging from 0 to 2.5 mm, which can be ascribed to the biological variation in response to treatment.^28^

FFAs are fixed to the teeth, and invariably, some amount of dental changes occur, and the total therapeutic change in any functional therapy is the result of a combination of skeletal and dental correction that takes place. The achievement of a class I molar relationship marked the end of the functional phase in this study. Dental changes observed in the present study were favorable towards class II correction, and upper molars and incisors moved backward; lower molars and incisors moved forward.

The upper incisor position (is/OLp) changed significantly from 83.5 mm to 79.6 mm (*P*< 0.001). The position of the upper incisor within the maxilla (is/OLp-ss/OLp) decreased from 11 to 9 mm (*P*= 0.006), suggesting a retroclination of upper incisors. Dental changes with the maxillary incisor were more pronounced compared to other studies.^8,12^ This can be attributed to the anchorage design consisting of total anchorage in the mandible with partial anchorage in the maxilla. The upper molar moved distally by 1.5 mm (*P*= 0.058).

Lower incisor position changes (in total and within the mandible), though favorable, were not consequential. Lower molar changes were highly pronounced (*P*< 0.001). The lower molar position within the lower jaw changed significantly (*P*= 0.002). Overall dental changes in the maxilla were more than the mandible, indicating a loss of anchor in the upper arch alone due to the anchorage design.

Molar relationship change (is/OLp-ii/OLp) and overjet (ms/OLp-mi/OLp) changes were highly significant. For molar and overjet correction, skeletal changes predominated with 61% and 63%, respectively. This favorable orthopedic outcome is due to the selection of patients in the pre-pubertal and circum-pubertal period. The dental changes accounted for 39% for molar correction and 36% for overjet correction. These findings are similar to the effect produced by the Herbst appliance.^25^

The anchorage design can also influence the degree of maxillary and mandibular dental changes. This appliance was designed to obtain full anchorage from the mandible and partial anchorage from the maxilla. Accordingly, more dental changes were observed in the maxilla than mandible, with more skeletal changes in the mandible. By varying the anchorage design, a custom-made appliance can be constructed, considering the phenotype of the malocclusion. This type of component approach by varying the number of teeth included is an advantage specific to FFA.

Anchorage can also be maximized with the help of miniscrews, thereby increasing the orthopedic effect.^28^ Pancherz^4^ stressed the importance of proper occlusal interdigitation as the key to post-treatment stability. Although the present study is short term, it showed that with correction of molar relationship at T2, posterior interdigitation was achieved in a few cases. This can be achieved only when the teeth are free to erupt without any occlusal coverage, which, in turn, depends on the design of the appliance. Johnston^29^ recounts that this interdigitation with functional correction “locks” the mandible to the maxilla. Hence during the post-functional period, the growth of the maxilla controls mandibular displacement, and both grow in unison, whereas in the functional phase, maxillary growth is restricted and mandibular growth is enhanced.

Limitations of this study include the small sample size because of its nature (preliminary) and intra-operator reliability. Although the Herbst appliance has been extensively researched, further studies are required to evaluate patients’ perception of treatment and experience with the Flip-Lock Herbst appliance.

The preliminary study on the Flip-Lock Herbst appliance showed favorable skeletal and dental changes. These changes were similar to those produced by the Herbst appliance in previous studies,^12,26,24^ with an added advantage of comfort and ease of lateral mandibular movements enabled by the ball joint type of connector. No ulcerations or injuries were noted in any of the patients. The appliance was both operator and patient-friendly.

## Conclusion

The Flip-Lock Herbst appliance was effective in correcting Angle’s class II division 1 malocclusion due to a retrognathic mandible. Both skeletal and dental changes occurred, with the former predominating (60:40).

## Authors’ Contributions

SRI and PKS: conceptualization and design of the study; SRI, MM, and PKS: data acquisition, analysis, and interpretation; SRI and PKS: drafting the work or revising it critically for important intellectual content. All authors have read and approved the final manuscript.

## Acknowledgments

The authors thank the statistician Mr. Boopathi for his assistance in this preliminary study.

## Competing Interests

The authors declare no conflict(s) of interest related to the publication of this work.

## Ethics Approval

The protocol of the study was approved by the Institutional Ethics Committee (ref. no. 0420/DE/2016).
